# A17 Amacrine Cells and Olfactory Granule Cells: Parallel Processors of Early Sensory Information

**DOI:** 10.3389/fncel.2020.600537

**Published:** 2020-11-05

**Authors:** Veronica Egger, Jeffrey S. Diamond

**Affiliations:** ^1^Department of Neurophysiology, Institute of Zoology, Universität Regensburg, Regensburg, Germany; ^2^Synaptic Physiology Section, National Institute of Neurological Disorders and Stroke, National Institutes of Health, Bethesda, MD, United States

**Keywords:** retina, olfactory bulb, reciprocal synapse, inhibition, parallel processing, local feedback, sensory processing

## Abstract

Neurons typically receive synaptic input in their dendritic arbor, integrate inputs in their soma, and send output action potentials through their axon, following Cajal’s law of dynamic polarization. Two notable exceptions are retinal amacrine cells and olfactory granule cells (GCs), which flout Cajal’s edict by providing synaptic output from the same dendrites that collect synaptic input. Amacrine cells, a diverse cell class comprising >60 subtypes, employ various dendritic input/output strategies, but A17 amacrine cells (A17s) in particular share further interesting functional characteristics with GCs: both receive excitatory synaptic input from neurons in the primary glutamatergic pathway and return immediate, reciprocal feedback *via* GABAergic inhibitory synapses to the same synaptic terminals that provided input. Both neurons thereby process signals locally within their dendrites, shaping many parallels, signaling pathways independently. The similarities between A17s and GCs cast into relief striking differences that may indicate distinct processing roles within their respective circuits: First, they employ partially dissimilar molecular mechanisms to transform excitatory input into inhibitory output; second, GCs fire action potentials, whereas A17s do not. Third, GC signals may be influenced by cortical feedback, whereas the mammalian retina receives no such retrograde input. Finally, A17s constitute just one subtype within a diverse class that is specialized in a particular task, whereas the more homogeneous GCs may play more diverse signaling roles *via* multiple processing modes. Here, we review these analogies and distinctions between A17 amacrine cells and granule cells, hoping to gain further insight into the operating principles of these two sensory circuits.

## Circuitry

**Figure 1:**
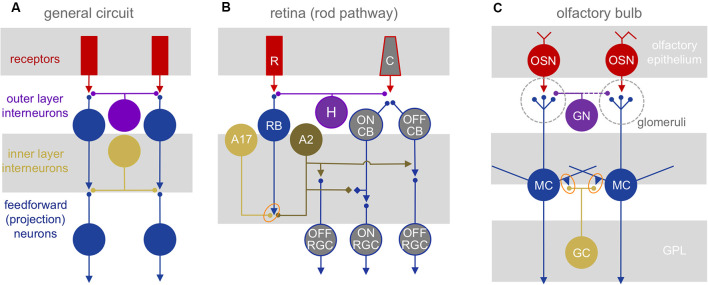
Neuronal circuit architectures. **(A)** General network elements common to the retina and olfactory bulb: two layers of inhibitory interactions are mediated by segregated subsets of local interneurons. **(B)** Mammalian retinal circuitry, with the rod pathway highlighted. Rod photoreceptors (R) contact rod bipolar cells (RB), which in turn contact A17 and A2 amacrine cells. The A2 relays the ON signal from rod bipolar cells to the cone pathway. C, cone photoreceptor; H, horizontal cell; CB, cone bipolar cell; RGC, retinal ganglion cell. Orange oval highlights reciprocal synapse between RB and A17. **(C)** Olfactory bulb circuitry. Olfactory sensory neurons (OSN) project into glomeruli, contacting glomerular neurons (GN) and the apical dendritic tufts of mitral (and tufted) cells (MC). The MC lateral dendrites form reciprocal synapses (orange oval) with granule cell (GC) and other interneuron dendrites. GN, glomerular neurons.

Both the retina and olfactory bulb are strictly layered early sensory processing areas with myriad interneuron types that provide local and lateral interactions between sensory input channels (Figure 1A). In the retina, these channels correspond to the local receptive fields of photoreceptors which transduce incident light from the visual world into a neural signal that is passed through glutamatergic synapses to bipolar cells and then onto the retinal projection neurons, the ganglion cells (RGCs; Figure 1B). In the olfactory bulb, the channels correspond to the glomerular modules that are innervated exclusively by one of several 100s-2,000 olfactory receptor neuron types—each expressing a distinct olfactory receptor—in the nose. These receptor neurons are excited by volatile odorants and pass a glutamatergic signal to mitral and tufted cells (MTCs), the bulbar projection neurons (Figure 1C).

While the bulb contains no direct neuronal analog to retinal bipolar cells, we propose that the highly excitable dendritic tufts of MTCs, which can produce regenerative signals on their own (Chen et al., 1997; Yuan and Knöpfel, 2006) may represent their counterparts. In both systems, neighboring excitatory projection neurons (RGCs and MTCs) typically are not directly interconnected *via* chemical or electrical synapses, although MTC tufts within the glomeruli may interact *via* glutamate spillover between synapses or electrical coupling (Schoppa and Westbrook, 2001, 2002).

Signals in both primary sensory pathways are sculpted in two stages by distinct, laterally structured inhibitory networks: in the outer retina, horizontal cells feed back onto photoreceptors to craft center-surround receptive fields (Baylor et al., 1971). In the outer layer of the bulb, a diverse set of glomerular neurons (GN) mediates intra- and interglomerular interactions between the sensory axons and the dendritic tufts of MTCs (reviewed in Wachowiak and Shipley, 2006; Burton, 2017). In the inner retina, amacrine cells (ACs) provide feedback and feedforward inhibition to both bipolar cells and/or RGCs, and in the inner bulb, MTCs interact with local interneurons that consist mostly of granule cells, although other interneuron subtypes contribute substantially to odor processing (Toida et al., 1994; Lepousez et al., 2010; Huang et al., 2013; Kato et al., 2013; Miyamichi et al., 2013). Both GCs and some ACs make GABAergic feedback inhibitory synapses onto the same synaptic terminals that provide them excitatory input (Rall et al., 1966; Kolb and Famiglietti, 1974). Both GCs and many ACs are connected by gap junctions (Reyher et al., 1991; Vaney, 1994; Menger and Wässle, 2000). GCs also receive powerful glutamatergic, centrifugal inputs (Price and Powell, 1970c; Balu et al., 2007; Pressler and Strowbridge, 2017), whereas ACs do not.

ACs are molecularly and morphologically diverse: 63 molecularly defined subtypes also differ concerning dendritic arbor size, branching patterns, projection depth in the inner plexiform layer (IPL), and synaptic partners (Diamond, 2017; Yan et al., 2020). GC subtypes are less well characterized; the current count of six morphological subtypes likely underestimates their molecular diversity, especially considering differences between GCs born neonatally and during adult neurogenesis (Breton-Provencher and Saghatelyan, 2012; Nagayama et al., 2014; Takahashi et al., 2018); there is no adult neurogenesis of ACs. Greater interneuron diversity in the retina may be required to support more parallel output channels: the number of distinct RGC types (currently Baden et al., 2016; Rheaume et al., 2018; Laboissonniere et al., 2019; Tran et al., 2019) may exceed that of MTC projection neurons by an order of magnitude (Imamura et al., 2020).

GCs make all of their synaptic outputs from apical dendritic spines that receive excitatory inputs primarily from the lateral dendrites of MTCs (Price and Powell, 1970b; Naritsuka et al., 2009). Because this prominent feature equips GCs for parallel processing, we compare them here with A17 cells, the AC subtype that is most similar concerning synaptic interactions: A17 cells also perform local signal processing within reciprocal synapses that are contained in dendritic varicosities from which they provide reciprocal feedback onto rod bipolar cells (RBCs; Chávez et al., 2006; Grimes et al., 2010). In the rod pathway which mediates night vision, A2 ACs relay RBC signals to the cone pathway (Famiglietti and Kolb, 1975; Pourcho and Goebel, 1985; Strettoi et al., 1992), whereas A17s interact exclusively with RBC terminals to modulate signal transfer to the A2s. Glutamatergic inputs from RBCs to A2s and A17s occur at “dyad” synapses in which each RBC active zone is apposed to two postsynaptic elements, usually one A2 and one A17. Individual GCs and A17s contain similar numbers of reciprocal synapses (150–200; Price and Powell, 1970a; Grimes et al., 2010; Geramita et al., 2016).

## Biophysical Characteristics

A17s and GCs exhibit distinctive morphological and membrane properties that enable them to provide reciprocal feedback inhibition in parallel through a large number of dendrodendritic synapses that can operate largely independently of one another within the same cell. They achieve these analogous goals using markedly different strategies. The similar outcomes highlight interesting parallels between the two systems, and the differences may provide insights into distinct computational requirements of different sensory circuits.

### Morphological Specializations Isolate Feedback Synapses

**Figure 2:**
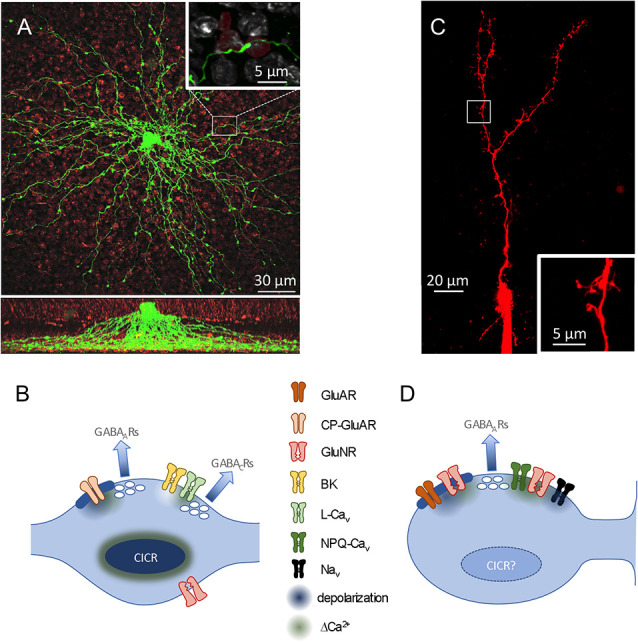
Dendritic and synaptic anatomy. **(A)** *Top*, confocal image (*z* projection) of an A17, filled with lucifer yellow. PKC-positive RBC terminals are red. *Inset*, Magnified view of a single RBC-A17synaptic contact. *Bottom*, Side view of 3D confocal stack showing A17 dendrites extending to the deepest part of the inner plexiform layer (IPL; modified from Grimes et al., 2015). **(B)** Schematic of reciprocal feedback synapses in an A17 dendritic varicosity. Two synapses are made onto the same presynaptic RBC and each contacts a distinct population of GABA receptors (Grimes et al., 2015). At one synapse, Ca^2+^ influx through GluARs triggers GABA release. **(C)** Two-photon scan (*z* projection) of an olfactory GC filled with Alexa 594 *via* a patch pipette. Note the large reciprocal spines in the distal region. **(D)** Schematic of reciprocal feedback synapse in a GC. Both Ca_v_s and GluNRs contribute Ca^2+^ to trigger GABA release. See text for further abbreviations.

The clearest morphological similarity between GCs and most ACs—the absence of an axon—was first pointed out more than a century ago by Ramón y Cajal (1911) and posed a counterpoint to his Law of Dynamic Polarization (Ramón y Cajal, 1891). Cajal typically identified distinct, segregated input and output regions to infer the direction of information flow through a neuron, but these clues are absent in GCs and most ACs. In A17s, for example, dozens of very thin (~130 nm diameter; Grimes et al., 2010) dendrites radiate, unbranched, from the soma like spokes on a wheel, extending deep into the inner plexiform layer and studded with varicosities (~1 μm diameter) at ~20 μm intervals (Zhang et al., 2002; Grimes et al., 2010; Figure 2A). Synaptic outputs are confined to the varicosities, which also receive synaptic inputs from RBCs (Nelson and Kolb, 1985). Models of this distinct morphology predicted that synaptic potentials would attenuate rapidly along the thin dendrites, possibly isolating neighboring varicosities from each other (Ellias and Stevens, 1980). Accordingly, imaging experiments showed that synaptic activation of single varicosities typically elicits only comparatively small Ca^2+^ signals in neighboring varicosities (Grimes et al., 2010).

GCs morphologically isolate their synapses differently. Each GC soma extends a large primary dendrite up to a branched arbor in the external plexiform layer (EPL) and only a few smaller dendrites down in the GC layer (Figure 2C). The dendrites are studded with prominent spines that receive synaptic input; in the EPL, a subset of particularly large spines (“gemmules;” Rall et al., 1966), deliver reciprocal synaptic outputs to MCs. GC apical dendrites are quite thick (~350–1,100 nm diameter; Rall and Shepherd, 1968) and, together with active conductances detailed below, enable membrane depolarizations to traverse the GC dendritic arbor (Egger et al., 2003). GC spine necks are long (nearly 2 μm) and thin (~230 nm diameter) and often contain mitochondria (Woolf et al., 1991) that likely increase their axial resistance. These spine necks reduce the extent to which signals in one spine influences neighboring spines, while also creating an electrotonically compact postsynaptic compartment that is more easily depolarized by synaptic and active conductances contained within (Bywalez et al., 2015).

### Active Dendritic Signaling

Active membrane conductances confer complex properties onto the dendrites of many neurons (London and Häusser, 2005). Voltage-gated sodium (Na_v_) channels in particular can initiate dendritic action potentials and/or propagate somatic action potentials retrogradely into the dendritic arbor (Stuart and Sakmann, 1994). In GCs, Na_v_s play both roles: large action potentials generated in the soma readily propagate into the EPL dendrites (Egger et al., 2003; Pressler and Strowbridge, 2019), and Na_v_s within the spines underlie local, regenerative events (“spine spikes”) that amplify postsynaptic potentials within the spine and allow a single MC input to elicit reciprocal feedback inhibition (Bywalez et al., 2015; Nunes and Kuner, 2018; Lage-Rupprecht et al., 2020). In most species, by contrast, A17s do not fire action potentials and (in rat) they express only relatively small Na_v_ conductances (~3nS; Grimes et al., 2010) that do not contribute significantly to dendritic signal propagation or reciprocal inhibition (Chávez et al., 2006, 2010; Grimes et al., 2010). Although A17 Na_v_ channels may underlie some heretofore unidentified local function, larger voltage-gated potassium (K_v_) conductances prevent Na_v_s from exerting more global influence (Menger and Wässle, 2000; Grimes et al., 2010). GC dendrites express A-type and delayed rectifier K_v_s (Hwang et al., 1993; Veh et al., 1995; Schoppa and Westbrook, 1999) that may limit interactions between spines, but they do not appear to influence signals from within reciprocal spines (Bywalez et al., 2015).

GCs employ distinct voltage-gated calcium (Ca_v_) channel subtypes for different tasks: T-type and L-type channels mediate Ca^2+^ influx into dendrites and spines (Egger et al., 2003, 2005; Pinato and Midtgaard, 2003, 2005; Pressler and Strowbridge, 2019; Müller and Egger, 2020), but N/P/Q-type Ca_v_s (along with NMDA receptors, see below) provide the Ca^2+^ required for synaptic release (Isaacson, 2001; Lage-Rupprecht et al., 2020). A17s, together with most non-spiking cells in the retinal circuitry (Pangrsic et al., 2018), express primarily L-type Ca_v_ channels (Grimes et al., 2010). In A17 varicosities, L-type Ca_v_s also activate large-conductance, Ca^2+^-activated K_v_ (BK) channels (Grimes et al., 2009), which regulate one component of neurotransmitter release (discussed in greater detail below). GCs also express BK channels (Isaacson and Murphy, 2001), although they do not appear to be present in the reciprocal spines (Bywalez et al., 2015).

Many neurons amplify intracellular Ca^2+^ signals *via* Ca^2+^-induced Ca^2+^ release (CICR) from intracellular stores (Parekh and Putney, 2005). CICR, a common signaling motif in amacrine cells (Warrier et al., 2005; Chávez and Diamond, 2008; Chávez et al., 2010), enhances Ca^2+^ signals and GABA release in A17 varicosities (Chávez et al., 2006; Grimes et al., 2009). CICR may contribute to postsynaptic Ca^2+^ signals in GC spines (Egger et al., 2005; Bywalez et al., 2015), but its effect on feedback GABA release remains unclear.

## Synaptic Characteristics

In both systems, circuit geometry conspires to limit the number of reciprocal synaptic connections between individual cell pairs. MTCs send their dendrites laterally across many orthogonally oriented GCs so that a particular GC rarely contacts an MTC more than once (Woolf et al., 1991). Varicosities on A17 dendrites are spaced 20 μm apart on average (Grimes et al., 2010), a distance greater than the breadth of an RBC synaptic terminal, again limiting most connected pairs to one synapse (Vaney, 1986; Zhang et al., 2002). In GCs and A17s, the presynaptic and postsynaptic machinery required for reciprocal feedback is co-localized within dendritic spines and varicosities, respectively, to facilitate direct coupling between synaptic input and output. GABA release from both cells relies conventionally on Ca^2+^ influx provided, at least in part, by unconventional sources.

In the mammalian retina, glutamate release from RBCs onto A17s activates primarily calcium-permeable AMPA receptors (GluARs; Hartveit, 1999; Chávez et al., 2006), although A17 varicosities also express extrasynaptic NMDA receptors (GluNRs; Zhou et al., 2016; Veruki et al., 2019; Figure 2B). Reciprocal feedback inhibition is mediated in RBC terminals by both GABA_A_Rs and GABA_C_Rs (Fletcher et al., 1998; Hartveit, 1999; Chávez et al., 2006, 2010; Eggers and Lukasiewicz, 2006; Frazao et al., 2007). A17 varicosities typically contain two feedback synapses that exhibit distinct characteristics and activate distinct GABAR populations (Fletcher et al., 1998; Grimes et al., 2015): one synapse, located closest to the presynaptic ribbon, contains GluARs and apposes primarily GABA_A_Rs, whereas a second, more distant synapse (~500 nm from the ribbon) expresses BK channels and apposes mostly GABA_C_Rs (Grimes et al., 2015). A17s express L-type Ca_v_s (Hartveit, 1999; Menger and Wässle, 2000; Grimes et al., 2009), but varicosities can release GABA in response to GluAR-mediated Ca^2+^ influx alone (Chávez et al., 2006). Stronger stimulation recruits the second component of release that is triggered by Ca_v_s, regulated by BK channels, and activates primarily GABA_C_Rs (Grimes et al., 2009, 2015). Distinct physiological roles for these two components of A17 feedback have yet to be identified.

MTC-released glutamate activates calcium-impermeable GluARs and GluNRs on GC spines (Trombley and Shepherd, 1992; Isaacson and Strowbridge, 1998; Schoppa et al., 1998; Isaacson, 2001; Figure 2D); GABA released from GCs activates GABA_A_Rs on MC dendrites (Nicoll, 1971). GluAR-mediated depolarization, amplified locally by Na_v_s in GC spines (Halabisky et al., 2000; Bywalez et al., 2015), activates (N and P/Q) Ca_v_s and relieves the Mg^2+^ block of GluNRs, enabling both to provide Ca^2+^ to trigger GABA release (Isaacson and Strowbridge, 1998; Schoppa et al., 1998; Halabisky et al., 2000; Isaacson, 2001), most likely *via* a cooperative mechanism (Lage-Rupprecht et al., 2020). Although GCs possess the machinery necessary to propagate and amplify depolarizations and Ca^2+^ signals in their dendrites and spines (Egger et al., 2003, 2005; Bywalez et al., 2015), it remains unclear whether GC spines can release GABA without direct glutamatergic input from MTCs.

In both systems, circuit anatomy, cellular biophysics, and synaptic characteristics ensure that feedback inhibitory input is largely decorrelated, possibly providing a low-noise inhibitory tone that may enhance the fidelity of feedforward signals.

## Sensory Processing

In sensory systems, the term “parallel processing” may refer to analogous computations duplicated simultaneously across some dimension, i.e., processing within each glomerular column, or the retinotopic representation of the visual world. Alternatively, it can refer to the task of encoding multiple stimulus features of an olfactory or visual stimulus. Here, we use the term to encompass both.

ACs diversify bipolar cell signals, thereby enabling contrast, orientation, motion and many other visual features to be encoded in dozens of parallel channels (Gollisch and Meister, 2010; Franke and Baden, 2017; Franke et al., 2017). Similarly, GCs have been proposed to contribute to decorrelation and gain control of MTC activity *via* the asynchronous release of GABA, so far mostly in the context of pattern separation required to distinguish between similar odorants (Friedrich et al., 2004; Abraham et al., 2010; Gschwend et al., 2015). It should be noted, however, that other interneurons may contribute, and the overall impact of GCs on MTC spiking frequency has been questioned (e.g., Fukunaga et al., 2014; Burton, 2017).

Spatial sensory maps are most apparent in the retina because neighboring cells and circuitry respond to similar regions of the visual world. The olfactory bulb may employ spatial chemotopic maps for subsets of odorants (e.g., Yokoi et al., 1995), but this may not constitute a general rule (reviewed by Murthy, 2011). In the retina, AC-mediated lateral inhibitory interactions underlie contrast enhancement and a more complex center-surround receptive field (e.g., Turner et al., 2018). Analogous roles have been proposed for GCs (Yokoi et al., 1995), but lateral interactions between MTCs were found later to be sparse and spatially dispersed (Fantana et al., 2008; Kim et al., 2011; Lehmann et al., 2016). Evidence suggests that reciprocal MTC-GC interactions underlie fast oscillations that pace MTC spiking (Lagier et al., 2004; Fukunaga et al., 2014) and, potentially, synchronize MTC activity across parallel active glomeruli. A17 reciprocal feedback inhibition has been proposed to increase the gain and sharpen the time course of transmission between RBCs and A2s, effects that may enhance the fidelity of signals in the rod pathway evoked by single photons (Grimes et al., 2015).

GC spines provide independent feedback inhibition (e.g., Isaacson and Strowbridge, 1998) in response to local unitary MTC input (Lage-Rupprecht et al., 2020), casting GCs, like A17s, as parallel processors. Accordingly, GC outputs are probably not activated solely by propagating dendritic action potentials, even though thresholds for such global signals are low (Lage-Rupprecht et al., 2020; Müller and Egger, 2020), although definitive experiments with paired MTC-GC recordings have remained elusive (Isaacson, 2001; Kato et al., 2013; Pressler and Strowbridge, 2017). These results suggest that GCs may be unable to inhibit MTCs in neighboring, quiescent glomeruli, a critical component of olfactory contrast enhancement, as observed *in vivo* (Fukunaga et al., 2014). Yet, GCs may mediate lateral interactions when they are activated more broadly, i.e., if neighboring glomeruli are activated simultaneously (Lage-Rupprecht et al., 2020). Moreover, centrifugal inputs onto GCs may drive GC spiking and facilitate lateral inhibitory signaling. Coincident activation of many A17 varicosities may enable them to interact (Grimes et al., 2010), although the required visual stimuli are unlikely to occur during scotopic (night) vision (Dunn et al., 2006).

## Author Contributions

VE and JD came up independently with the idea for this review, contributed to all parts of the manuscript, and approved the submitted version.

## Conflict of Interest

The authors declare that the research was conducted in the absence of any commercial or financial relationships that could be construed as a potential conflict of interest.
